# The Aging of Biomedical Research in the United States

**DOI:** 10.1371/journal.pone.0029738

**Published:** 2011-12-28

**Authors:** Kirstin R. W. Matthews, Kara M. Calhoun, Nathan Lo, Vivian Ho

**Affiliations:** 1 Science and Technology Policy Program, James A. Baker III Institute for Public Policy, Rice University, Houston, Texas, United States of America; 2 Department of Economics, Rice University, Houston, Texas, United States of America; Philipps-University Marburg, Germany

## Abstract

In the past 30 years, the average age of biomedical researchers has steadily increased. The average age of an investigator at the National Institutes of Health (NIH) rose from 39 to 51 between 1980 and 2008. The aging of the biomedical workforce was even more apparent when looking at first-time NIH grantees. The average age of a new investigator was 42 in 2008, compared to 36 in 1980. To determine if the rising barriers at NIH for entry in biomedical research might impact innovative ideas and research, we analyzed the research and publications of Nobel Prize winners from 1980 to 2010 to assess the age at which their pioneering research occurred. We established that in the 30-year period, 96 scientists won the Nobel Prize in medicine or chemistry for work related to biomedicine, and that their groundbreaking research was conducted at an average age of 41—one year younger than the average age of a new investigator at NIH. Furthermore, 78% of the Nobel Prize winners conducted their research before the age of 51, the average age of an NIH principal investigator. This suggested that limited access to NIH might inhibit research potential and novel projects, and could impact biomedicine and the next generation scientists in the United States.

## Introduction

With a budget of approximately $31 billion a year, the National Institutes of Health (NIH) (www.nih.gov) is one of the largest granting organizations in the world. President Barack Obama pledged in 2009 to “devote more than 3% of our GDP [gross domestic product] to research and development,” and to specifically “promote breakthroughs in energy and medicine [1].” Meanwhile, the US Congress, facing a massive budget deficit, is cutting discretionary spending. Thus, policymakers are now more interested than ever in how NIH money is being distributed. One major area of concern is the funding of young and first-time investigators.

NIH has been aware of the low funding rates of early-career scientists for years. As Dr. Elias Zerhouni, NIH director from 2002-2008, remarked in a talk at the James A. Baker III Institute for Public Policy, “you have to get a Nobel Prize before your first grant”—referring to Dr. David Baltimore, the 1975 Nobel Prize winner in medicine, who received the award at the age of 37, well under the average age of both an NIH principal investigator (PI) and a first-time NIH grantee [2].

**Figure 1:**
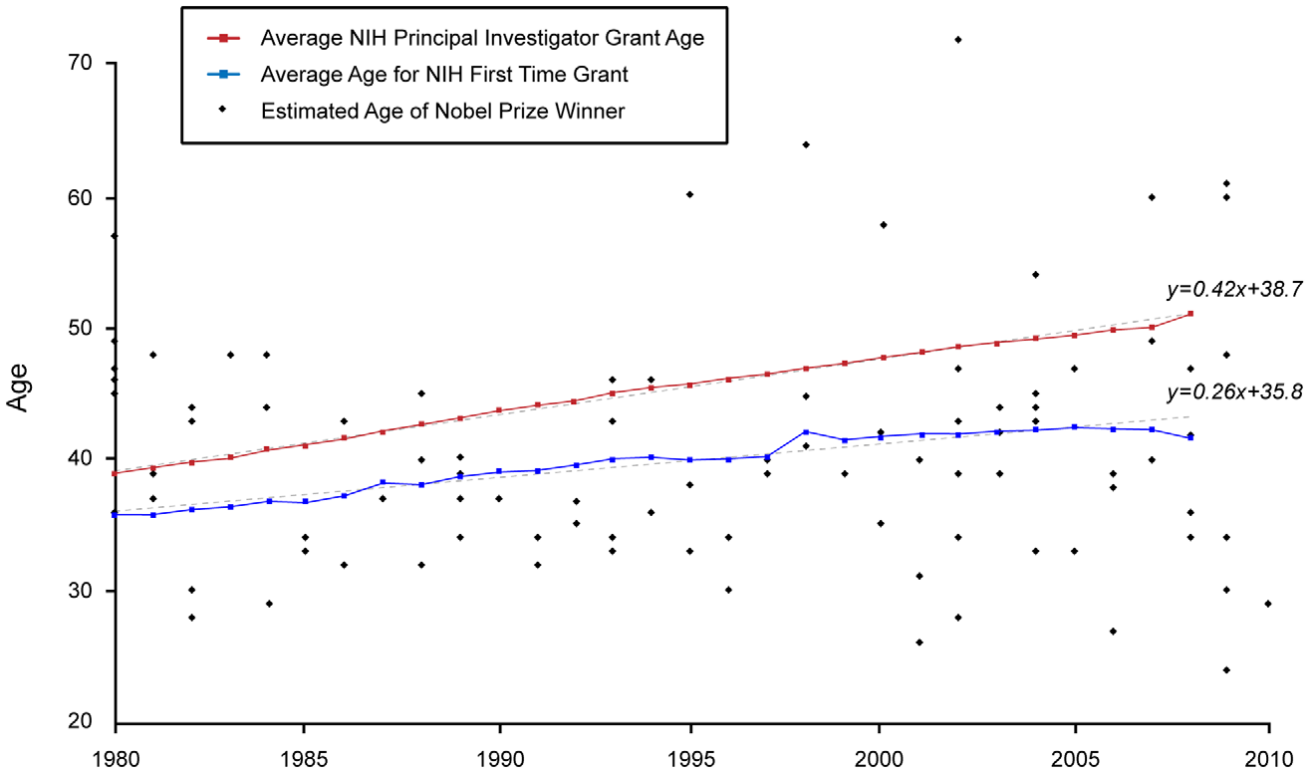
Age Comparison between NIH PIs and First-Time Recipients with Nobel Recipients, 1980- 2010. Since 1980, the average age of an NIH principal investigator (red) and a first-time grant recipient (blue) has steadily increased. The age of principal investigators is rising faster (with a slope of 0.42, p<.001) than first-time grant recipients (with a slope of 0.26, p<.001). Of the 96 Nobel laureates studied, 55 (57%) published their discoveries at an age under the average age for a first-time NIH grant (blue), and 75 (78%) published before the average age for a NIH grant (red). Ordinary least squares regression of the average age of Nobel prize winners on year of award indicated that there was no statistically significant increase in the age over time (p = 0.42). This suggested that investigators are publishing Nobel-worthy research at a younger age than a first-time NIH grantee or the average principal investigator.

The average age of a PI at NIH has increased at a steady pace from 39 in 1980 to 51 in 2008 (see Table 1) [3]. If this trend continues, it is predicted that the number of investigators over age 70 receiving NIH grants will surpass the number under 40 in 2020 [4]. The average age of first-time grantees has also increased in the past three decades (Figure 1). In 1980, the average age of a first-time grant recipient was 36. By 2008, this number had increased to 42. The age range of traditional investigator (R01-equivalent) grantees also increased from 24–67 in 1980 to 29–85 in 2008 [3], [5]. Now less than 5% of investigators are under the age of 37, the age of Dr. Baltimore when he received the Nobel Prize, compared to 36% in 1980. While the average age of the US population is steadily increasing, the NIH figures (51 years in 2008) were higher than the average age of the US labor force (41 in 2008) and statistically higher than the average age of US science and engineers faculty members (47 in 2006), even when adjusted to 2006 data (p<0.001) [6], [7].

**Table 1:** Range, Median and Averages of NIH PIs and First-time Recipients and Nobel Prize Winners.

		Range	Median	Average
New PI	1980	24 to 66	45	35.7
	2008	29 to 69	49	41.6
Ave PI	1980 PI	24 to 67	45.5	39.0
	2008PI	29 to 85	57	51.1
Nobel*	Ave	24 to 72	41	39.9

*Average of winners from 1980 to 2008.

With the increasing age of those obtaining funding from NIH, could the United States be losing out on innovative research and discouraging promising students from entering or continuing in science? Previous research suggested that the aging of the biomedical research sector will negatively impact productivity, as younger scientists are more likely to produce high-impact publications [8]–[12]. Wray argued that scientists are most productive at mid-career, which he defined as age 36 to 45, when they have access to more material and social resources [13], [14].

This study set out to assess when innovative biomedical research previously occurred by first establishing the age at which pioneering ideas of Nobel Prize winners arose. The Nobel Prize is awarded to scientists who have made the most significant discoveries in science—discoveries that withstand the “test of time.” These breakthroughs not only impact the scientists' respective fields, but also the advancement of other areas of science [15]. Furthermore, this prestigous award gives scientists public recognition and affirms their standing with their peers. The Nobel is a rare award; other awards are more limited in scope, are less consistently awarded (i.e., not annual) or, for the purposes of this study, do not have the database of information necessary to track the work being honored. For these reasons, we believed it was a good model to study.

We began our study of NIH aging trends by surveying the publications of scientists who won the Nobel Prize in medicine or chemistry for work related to biomedicine, and deduced the age at which the research occurred. Our results indicated that the majority of winners were under the average age of first-time NIH grant recipients when the Nobel-recognized research was conducted. This suggested that the NIH might be setting high barriers for entry into biomedical research, as demonstrated by the rising age of PIs and first-time grantees.

## Methods and Results

### Nobel Laureate Age

To ascertain the age at which Nobel laureates conducted their winning research, we first determined the project or idea that led to the prize and matched it to a corresponding publication. The list of recipients, obtained directly from the Nobel website, www.nobelprize.org, included all winners from 1980 to 2010 whose work was related to biomedical research. This consisted of all medicine and physiology winners and a subset of chemistry winners whose research involved DNA, RNA, or proteins. Using the description of the research and press releases from the Nobel website, we deduced the research topic and searched PubMed, the online database of biomedical research (www.pubmed.org), to find the seminal publication. We established either the specific publication for which the award was granted (as was the case for the 2005 award for *Helicobacter pylori* to Drs. Barry J. Marshall and J. Robin Warren) or estimated when the researcher first published his or her pioneering work (as was the case for the 2003 award to Drs. Paul C. Lauterbur and Peter Mansfield for magnetic resonance imaging [MRI]). The publication year determined the age at which each winner conducted innovative research leading to his or her award. This methodology is similar to previous studies on Nobel laureates [16], [17]. The technique might overestimate the age of the investigator slightly since it relies on the date of publication, which can follow the actual experiments by months or years.

**Figure 2:**
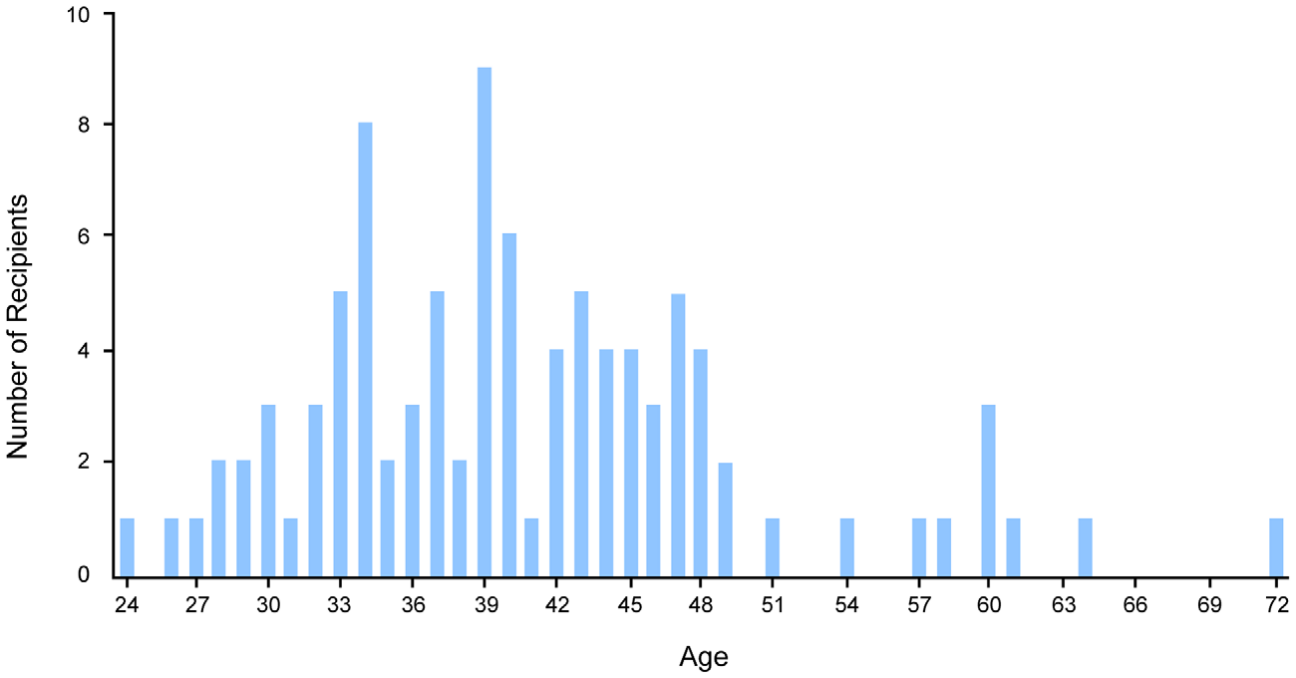
Estimated Age Distribution of Biomedicine-related Nobel Recipients, 1980-2010. The estimated age distribution of the 96 laureates at time of publication of their Nobel Prize research in biomedicine ranged from 24 to 72. The average age of recipients was 41 and the majority (61%) were under the age of 42, indicating that the distribution was not symmetric and was skewed toward younger investigators.

In the 30-year period studied (1980 to 2010), 96 people with biomedical research interests were awarded the Nobel Prize. This seemingly high total resulted from the naming of multiple winners in each category in most years (Figure 1). Of the 96 Nobel Prize recipients, 70 were in the field of medicine and 26 were in chemistry. The publication age for the entire group ranged from 24 to 72 years old, with an average age of 41 and a median age of 39.5 (Figure 2 and Table 1). Our study also showed that 61% of these winners published their first research related to the Nobel Prize work before the age of 42, the average age of first-time NIH grant recipients in 2008. In addition, 57% were below the average age of a first-time NIH grantee in the year they published their groundbreaking work. For example, Dr. Carol W. Greider received the Nobel Prize in 2009 for work she started publishing as an approximately 24-year-old graduate student in 1985, when the average age for a first-time NIH grant recipient was 36.8 years old. Furthermore, 78% of the Nobel winners published their work at an age that was younger than the overall average age for an NIH PI (Figure 1). Ordinary least squares regression of the average age of Nobel Prize winners on year of award indicated that there was no statistically significant increase in age over time (p = 0.42).

### Nobel Laureate Funding

Once the list of laureates and their publications was generated, the funding sources for US recipients (based on the address listed on the publication) were determined. Of the 96 Nobel Prize winners, 59 recipients (62% of the group) were from the United States. Each US publication was examined for specific funding information in the acknowledgment section of the laureate's paper. If possible, the NIH RePORT grant information database (http://projectreporter.nih.gov) was searched to verify the grant and topic. However, the database is limited to grants awarded after 1986, and many projects were conducted prior to this date.

To confirm previous findings and obtain missing information, we emailed each of the US laureates, when possible, asking the question, “Could you confirm if you were being funded through an NIH grant during the period of time you were conducting the work that led to your Nobel Prize?” Some laureates were not emailed because an address could not be obtained or they died prior to the survey. The information obtained from the email responses was compared to previously collected data to determine the status of NIH funding at the time of their groundbreaking work; the results were consistent with previous findings.

From the US group of laureates, we emailed 36 individuals and obtained responses from 23. Using the information obtained from the survey, their respective papers, and the NIH RePORT site, we were able to verify the funding source for 76.3% of the US Nobel laureates (45 of the 59). Sixteen (more than one-third) of the 45 American Nobel laureates did not receive funding from the NIH for the early scientific work that won them the prize. The work of seven of these recipients was funded through NIH grants, with others listed as the PI. For instance, Dr. Carol W. Greider's early work was funded under Dr. Elizabeth H. Blackburn, her Ph.D. adviser. The remaining recipients were funded through private institutions—PEW, the March of Dimes, the American Cancer Society, the American Heart Association, their university or a company—or other governmental agencies, specifically the Atomic Energy Commission or the US Public Health Service. These results are similar to findings from other researchers who looked at Nobel funding from 2000 to 2008 in the fields of chemistry, physics, and medicine [18].

## Discussion

As scientific research becomes more competitive, it is crucial to fund the most promising research and promote innovative and creative thinking. In this paper, we used the Nobel Prize as a measure for innovation or innovative thinking to try to estimate when novel and revolutionary ideas are formed, specifically in biomedical research. This study suggested that Nobel Prize winners conducted their groundbreaking research at an average age that was lower than the average age of an NIH PI or a first-time NIH grantee. Our data also suggested that while innovative research can occur at any age (the Nobel researchers surveyed ranged in age from 24 to 72 years), investigators under 40 years old seemed to dominate the projects. While many of the young investigators collaborated with more senior scientists, the fact that they were singled out by the Nobel committee indicated their personal contribution was great, and the field may not have advanced without them.

The peer-review process can be the best way to determine good projects, but it can also be conservative and risk-averse. This can harms both high-risk, high-impact ideas as well as new investigators who have not established a track record. First-time investigators are historically funded at lower rates due to their perceived higher risk. In 2006, 14.8% of proposals from first-time investigators received funding, compared to 17.5% for previously funded investigators [3].

There are also other possible reasons for the lower average age of Nobel winners compared to first-time grantees. Young researchers might have fewer management obligations and grant writing pressures than senior faculty members, and might therefore spend more time in the lab on experiments. They also have access to reagents and equipment through their mentors, which might be missing in later stages of their careers. They could also benefit from associating with a mature PI and receiving increased mentoring.

In the limited survey of Nobel Prize winners, we found that in several cases, the NIH did not fund the researchers directly for their work leading to the award. Responses to our emails uncovered multiple grant proposal rejections due to the researcher's inexperience or the peer reviewer's reluctance to fund high-risk projects. By raising barriers to funding, the NIH could be stifling innovative projects by deterring young scientists from entering the field. The NIH budget has been boosted nearly tenfold in the past 30 years, indicating that more resources are available and could be allocated differently to support early-career scientists (www.nih.gov).

The American Academy of Arts and Sciences specifically addressed high-risk, high-reward, and early-career investigators in the publication “Advancing Research in Science and Engineering: Investing in Early-Career Scientists and High-Risk, High-Reward Research [19].” The report recommends providing seed funding that allows early-career investigators to explore novel projects without the requirement of preliminary results and increasing funding rates overall. It also mentions that low grant success rates can further discourage scientists from pursuing high-risk projects [20].

**Figure 3:**
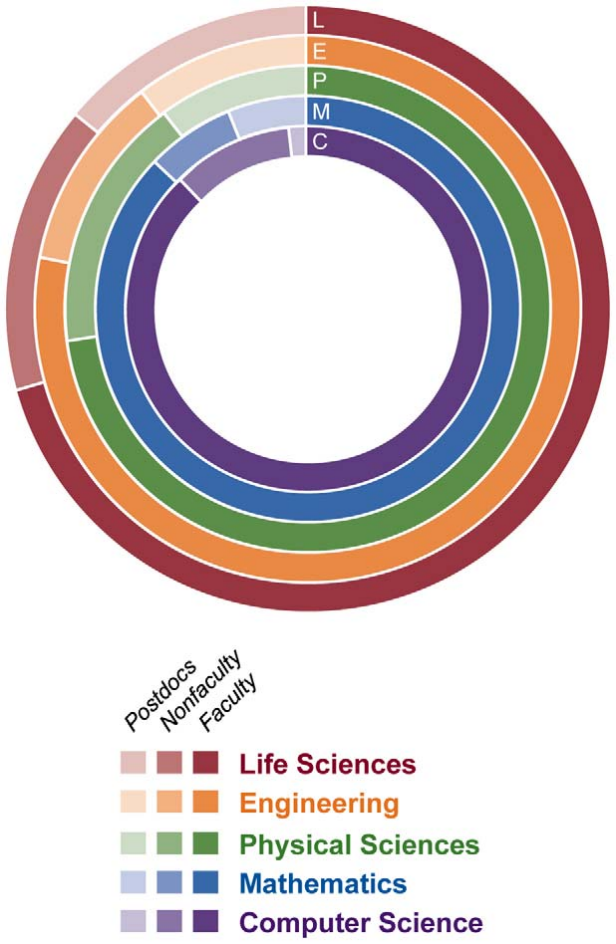
Science and Engineering Doctorate Holders Employed in Academia in 2006. The proportion of full-time faculty (dark shade) was lowest for the life sciences (red) across all science and engineering fields in 2006. The life sciences also had the highest percentage of postdoctoral fellows (lightest shade) across all the fields. This was in contrast to computer science (purple), which had the highest proportion of full-time faculty and lowest proportion of postdoctoral fellows. SOURCE: National Science Foundation, Science and Engineering Indicators 2010.

**Figure 4:**
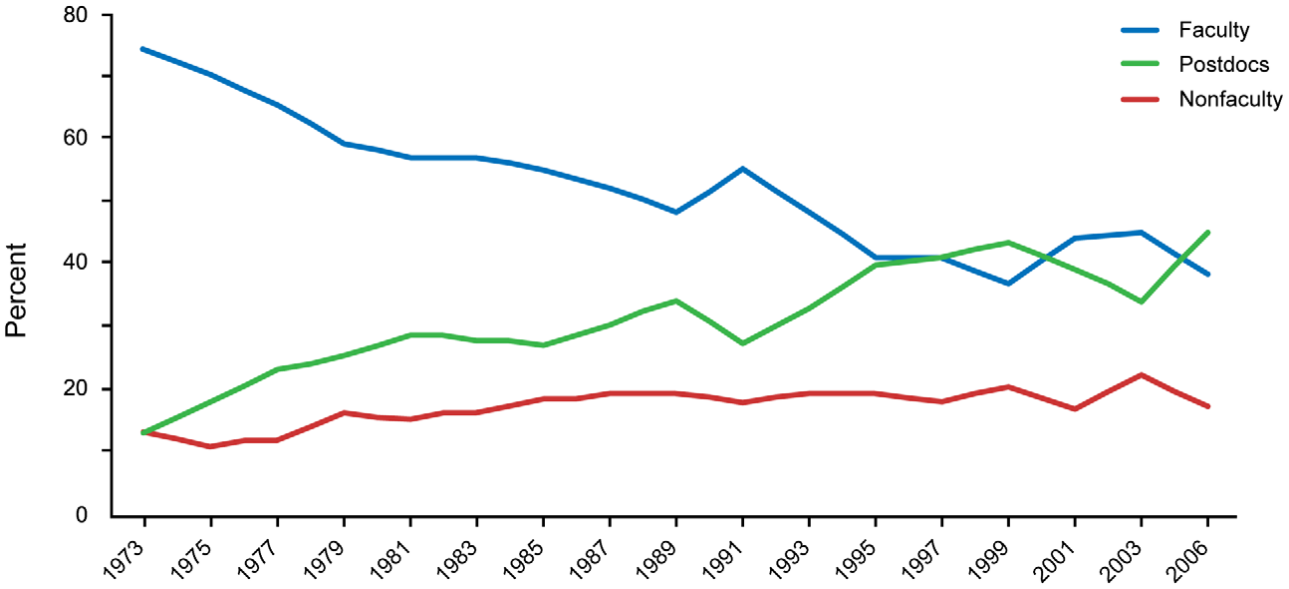
Number of Positions in the Biological Sciences, 1981–2006. The number of full-time faculty (blue) in the biological sciences has decreased between 1981 and 2006, while the number of postdoctoral fellows (green) has increased, and number of nonfaculty full-time positions (red) has remained relatively steady. Faculty included full-time full, associate, and assistant professors plus instructors. Nonfaculty positions included research associates, adjunct appointments, lecturers, and administrative positions. SOURCE: National Science Foundation, Science and Engineering Indicators 2010.

**Figure 5:**
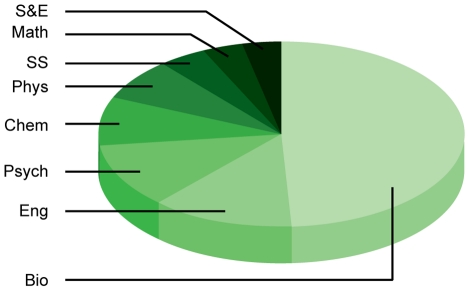
Science and Engineering Postdoctoral Fellows, by Field in 2005. Biological sciences (lightest shade) represented the overwhelming majority of postdoctoral fellows in 2005 (49.1%) compared to other sciences. SOURCE: National Science Foundation, Science and Engineering Indicators 2010.

Difficulties obtaining funding can negatively impact the career choices of young scientists, particularly in the biological sciences. Researchers in the biological sciences are waiting longer for independence or to start their own research projects than in other scientific fields [21]–[23]. In 1973, 55% of US doctorates in bioscience secured tenure track positions within six years of completing their Ph.D.s; by 2006, this number dropped to 15% [24]. After comparing faculty, nonfaculty, and postdoctoral researchers in different areas of science and engineering, the life sciences stand out as having the lowest percentage of Ph.D.s employed in full-time faculty positions and the highest percentage of postdoctoral fellows at 13.4% (Figure 3) [24]. Data from the National Science Foundation (NSF) also indicates a growing proportion of US Ph.D.s in postdoctoral and nontenured positions compared to 30 years ago (Figure 4) [24]. The number of medical and other life sciences postdoctoral positions increased 2.5 times, from 5,200 in 1981 to 14,450 in 2006, while the number of full-time faculty positions in the biological sciences only increased 1.6 times, from 40,900 in 1981 to 64,500 in 2006 [24]. This suggests that potential talent vastly exceeds the number of faculty positions available for young working scientists. Overall, close to half of all postdoctoral positions are in the biological sciences (Figure 5) [24]. In contrast, computer science, a field known for innovation, has the highest percentage of Ph.D.s in full-time faculty positions and only 1.7% employed as postdoctoral fellows (Figure 3).

The lack of funding and positions also indicates that the current American Ph.D. system might need to be reevaluated. As the 2011 *Nature* article “Fix the Ph.D.” points out, “[e]xceptionally bright science Ph.D. holders from elite academic institutions are slogging through five or ten years of poorly paid postdoctoral studies, slowly becoming disillusioned by the ruthless and often fruitless fight for a permanent academic position” [25]. Even outside the biological sciences, the number of Ph.D.s in all sciences worldwide has increased 40% between 1998 and 2008; in the United States, many believe that this has led to a surplus of science Ph.D.s competing for a limited number of academic positions [26]–[27]. This issue is amplified in the life sciences, where growth has been the largest and is coupled, as mentioned previously, with slower increases in the number of scientists receiving tenure. Although many are turning to industry, the number of positions available is not adequate to compensate for the lack of tenure-track positions; graduate schools have not limited enrollment accordingly or offered the training needed for alternative careers [28].

In response to the rising average age of first-time investigators, over the past three years the NIH has begun to emphasize, through the Office of the Director, the importance of finding new ways to support young scientists and improve their chances to obtain funding. Before leaving NIH, Dr. Zerhouni announced a series of policies intended for new and early-stage researchers, including grants specifically for new investigators and refining the peer review process to help reverse the bias against first-time grantees [3], [29]. Current NIH director Dr. Francis Collins has continued to stress that “we must liberate our brightest minds to pursue high-risk, high-reward ideas during their most creative years” [30]. To reduce barriers, he created a task force—chaired by Dr. Shirley Tilghman, president of Princeton University—charged with developing a model for creating a sustainable and diverse biomedical workforce [31]. As a result of these efforts, the NIH has shown an increase in new investigator grants. New investigators as a percentage of grants decreased from 33.4% in 1980 to 23.9% in 2006, but recovered to 29.5% in 2009 after the new NIH policy was implemented. Success rates have also improved, with 18% of established investigators versus 17% of first-time applicants receiving funding in 2010 [32].

Many of the new investigator grants were obtained through exceptions, such as when a program officer chose a grant proposal with a lower ranking and awarded it funding over other proposals with better scores. In 2007, 18.5% of funded RO1 applications, NIH's traditional investigator grant, were exceptions, compared to 9.7% in 2003 [33]. The increase in exceptions has caused some scientists to worry that preferential treatment is being given to some researchers at the expense of more senior investigators, and in the future, these new investigators will be unable to compete at the same level as others who did not get preferential treatment [34]. However, NIH administration believes that the exceptions were made by experts in their respective fields, improve an imbalance in the system, and do not need additional monitoring or oversight.

To prevent losing bright and talented young scientists, policymakers need to continue to encourage NIH investment in new investigators through increased funding of their research and by extending more offers of faculty positions at academic institutions [35]. Furthermore, attention should be paid to how Ph.D. programs are managed, including monitoring the number of students admitted each year to more effectively match the supply with the demand for jobs [36]. Additionally, it is imperative that NIH continues to stress the importance of supporting early-career scientists. Ultimately, the NIH is a public entity funded by taxpayers. The agency is responsible for demonstrating the value of the sponsored research. Its mission is to support incremental basic research for “fundamental knowledge about the nature and behavior of living systems” as well as more high-reward and applied projects for “the applications of knowledge to enhance health, lengthen life, and reduce the burdens of illness and disability” [37].

Although our research cannot determine if the rise in the average age of new investigators or PIs will directly affect future innovation or the proportion of US recipients awarded the Nobel Prize, we do believe it could influence the number of scientists entering and staying in biomedical research positions in academia. To further investigate the impact of NIH's aging trend, we believe further research should be conducted to determine the number of scientists leaving the field in early-career stages (i.e., between obtaining a Ph.D. and tenure). In addition, a careful survey of the impact of NIH's new policy granting exceptions for new investigators should be pursued to ascertain if these researchers are more, less, or equally as successful as their colleagues in future rounds of funding, when privileged status is not granted.

If nothing is done to reverse the rising age of PIs and first-time grantees, the scientific community could lose a generation of researchers, leading to an unsustainable biomedical research infrastructure and a dearth of talent participating in NIH-funded projects in the near future. In approximately 20 years, this gap will be more apparent as senior leadership enters retirement. Without change, biomedical research will be poised to falter as the next generation of scientists and innovations fails to emerge. Increasing the commitment to fund young scientists and providing career advancement opportunities will help ensure a steady flow of ingenuity for years to come.
